# ADHD traits, stigma, and well-being: A community study among Hungarian adolescents and young adults

**DOI:** 10.1192/j.eurpsy.2026.10235

**Published:** 2026-07-31

**Authors:** K. Vajsz, M. Miklosi, L. R. Paulina, D. Pető, B. Juhász, X. Gonda

**Affiliations:** 1 Department of Psychiatry and Psychotherapy, Semmelweis University; 2 Centre of Mental Health, Heim Pál National Pediatric Institute; 3 Institute of Psychology, Eötvös Loránd University, Budapest, Hungary

## Abstract

**Introduction:**

Beyond cognitive and behavioral difficulties, Attention-Deficit/Hyperactivity Disorder (ADHD) is often accompanied by stigma, including negative public attitudes, self-stigmatization, and poor mental health literacy. Such factors may compromise well-being during adolescence and young adulthood, a critical period for identity formation.

**Objectives:**

This study investigated the associations between ADHD-related traits, stigma, and well-being in Hungarian adolescents and young adults, focusing on how inattention and hyperactivity/impulsivity traits relate to emotional, social, and psychological well-being, and whether stigma components act as mediators.

**Methods:**

A cross-sectional community sample (N = 1295, age 14-25) completed validated measures: SWAN (Strengths and Weaknesses of ADHD Symptoms and Normal Behavior Scale), MHC-SF (Mental Health Continuum - Short Form), ASQ (ADHD Stigma Questionnaire), RIBS (Reported and Intended Behaviour Scale), and MAKS (Mental Health Knowledge Schedule). Correlations, linear regressions, and mediation analyses were performed.

**Results:**

Inattention traits were negatively associated with all well-being domains (r = 0.27-0.41, p < 0.001), while hyperactivity/impulsivity showed weaker but significant associations (r = 0.18-0.23, p < 0.001). In multiple regression models, inattention (β = -0.32, p < 0.001) and mental health knowledge (β = 0.12, p < 0.001) emerged as significant predictors of psychological well-being, with internalized stigma (ASQ negative self-image; β = -0.13, p < 0.001) exerting an additional negative effect. Emotional and social well-being were significantly predicted by inattention (β = -0.09 to -0.27, p < 0.01) and mental health knowledge (β = 0.09-0.27, p < 0.01). The final model predicting psychological well-being explained 17.5% of the variance (Adj. R² = 0.175, F(3,694) = 50.124, p < 0.001). Mediation analyses revealed limited but significant indirect effects of inattention on emotional well-being through stigma and knowledge.

**Image 1:**
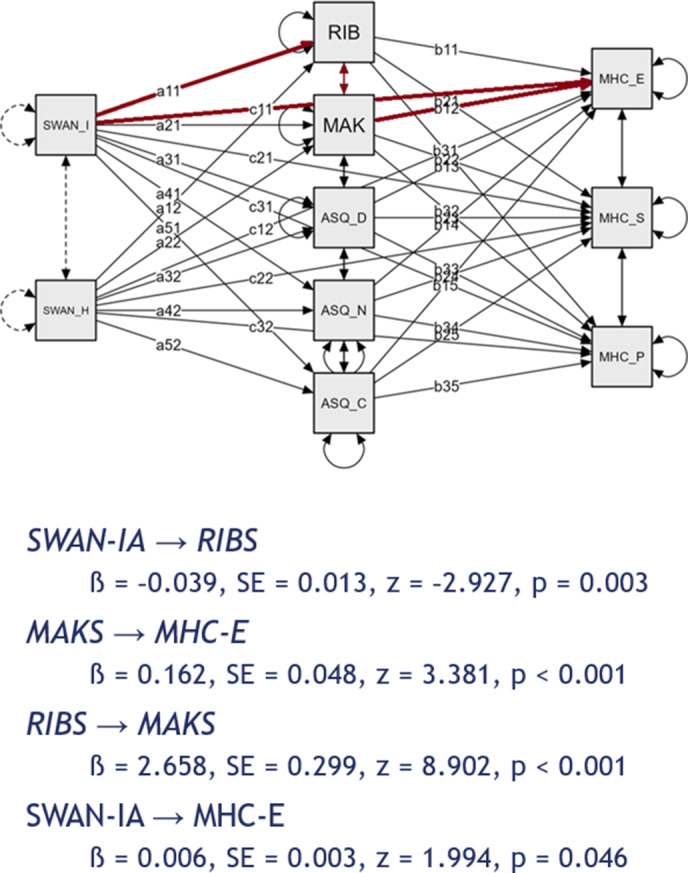
Image 1: Long description.

**Conclusions:**

ADHD-related inattention traits and mental health literacy are key determinants of youth well-being, while internalized ADHD-related stigma directly undermines eudaimonic well-being, which is closely related to good functioning and self-realization. Findings underscore the importance of psychoeducation and stigma-reduction efforts, even in non-clinical populations with elevated ADHD traits.

**Disclosure of Interest:**

None Declared

## Long descriptions

### Image 1: Long description

The top panel is a path diagram with three vertical columns of nodes.

Left column nodes: S W A N underscore I and S W A N underscore H, connected by a dashed double-headed arrow.

Middle column nodes from top to bottom: R I B, M A K, A S Q underscore D, A S Q underscore N, and A S Q underscore C. R I B and M A K are connected by a red double-headed arrow.

Right column nodes from top to bottom: M H C underscore E, M H C underscore S, and M H C underscore P.

Arrows originate from the left column to the middle and right columns, and from the middle column to the right column. Notable red paths include S W A N underscore I to R I B (labeled a 1 1), S W A N underscore I to M A K (labeled a 2 1), and M A K to M H C underscore E (labeled b 1 2). Other paths are labeled with alphanumeric codes like a, b, and c followed by digits.

The bottom panel lists four statistical paths:

1. S W A N minus I A yields R I B S: beta = -0.039, S E = 0.013, z = -2.927, p = 0.003.

2. M A K S yields M H C minus E: beta = 0.162, S E = 0.048, z = 3.381, p < 0.001.

3. R I B S yields M A K S: beta = 2.658, S E = 0.299, z = 8.902, p < 0.001.

4. S W A N minus I A yields M H C minus E: beta = 0.006, S E = 0.003, z = 1.994, p = 0.046.

Navigate back to Image 1:.

